# Irritable Bowel Syndrome and Neurological Deficiencies: Is There A Relationship? The Possible Relevance of the Oxidative Stress Status

**DOI:** 10.3390/medicina56040175

**Published:** 2020-04-13

**Authors:** Ioana-Miruna Balmus, Alin Ciobica, Roxana Cojocariu, Alina-Costina Luca, Lucian Gorgan

**Affiliations:** 1 Department of Interdisciplinary Research in Science, “Alexandru Ioan Cuza” University of Iasi, Carol I Avenue, No. 11, 700506 Iași, Romania; balmus.ioanamiruna@yahoo.com; 2 Department of Research, Faculty of Biology, “Alexandru Ioan Cuza” University of Iasi, Carol I Avenue, 20A, 700506 Iași, Romania; 3 Department of Biology, Faculty of Biology, “Alexandru Ioan Cuza” University of Iasi, Carol I Avenue, 20A, 700506 Iași, Romania; roxana_20_2006@yahoo.com (R.C.); lucian.gorgan@uaic.ro (L.G.); 4 Faculty of Medicine, “Gr. T. Popa” University of Medicine and Pharmacy, 16th University Street, 700115 Iași, Romania

**Keywords:** irritable bowel syndrome, neurological deficiencies, oxidative stress

## Abstract

*Background:* Irritable bowel syndrome (IBS) is one of the most common functional gastrointestinal disorders, exhibiting complex and controversial pathological features. Both oxidative stress and inflammation-related reactive oxygen species production may be involved in IBS pathological development. Thus, we focused on several aspects regarding the causes of oxidative stress occurrence in IBS. Additionally, in the molecular context of oxidative changes, we tried to discuss these possible neurological implications in IBS. *Methods*: The literature search included the main available databases (e.g., ScienceDirect, Pubmed/Medline, Embase, and Google Scholar). Articles in the English language were taken into consideration. Our screening was conducted based on several words such as “irritable bowel syndrome”, “gut brain axis”, “oxidative stress”, “neuroendocrine”, and combinations. *Results*: While no consistent evidence suggests clear pathway mechanisms, it seems that the inflammatory response may also be relevant in IBS. The mild implication of oxidative stress in IBS has been described through clinical studies and some animal models, revealing changes in the main markers such as antioxidant status and peroxidation markers. Moreover, it seems that the neurological structures involved in the brain-gut axis may be affected in IBS rather than the local gut tissue and functionality. Due to a gut-brain axis bidirectional communication error, a correlation between neurological impairment, emotional over-responsiveness, mild inflammatory patterns, and oxidative stress can be suggested. *Conclusions*: Therefore, there is a possible correlation between neurological impairment, emotional over-responsiveness, mild inflammatory patterns, and oxidative stress that are not followed by tissue destruction in IBS patients. Moreover, it is not yet clear whether oxidative stress, inflammation, or neurological impairments are key determinants or in which way these three interact in IBS pathology. However, the conditions in which oxidative imbalances occur may be an interesting research lead in order to find possible explanations for IBS development.

## 1. Introduction

Reactive oxygen species (ROS) and reactive nitrogen species (RNS) are the most common and abundant small active molecules in normal metabolism. Although they are essential for physiological functions, the disruption of pathways involving ROS or RNS can lead to powerful death signals [1]. In this manner, partially reduced molecular oxygen species that are continuously produced by cellular respiration, enzymatic reactions, and the immune response, which are extremely reactive due to unpaired valence-shell electrons, may be involved in key regulation points in cellular metabolism, as well as in survival/death signaling [2]. Thus, any imbalance between ROS production and neutralization leads to oxidative stress. Many studies link oxidative stress with several widely known diseases and pathological processes such as abnormal aging, cardiovascular and renal injuries, cancer, and various neuropathies and neurological syndromes, such as Alzheimer’s disease [3]. 

Normal cellular metabolism is generally known to include powerful toxic metabolite neutralization systems. ROS, which are the normal byproducts of oxidative pathways, seem to present no exception to this rule [4]. Apart from the toxic roles characteristic of high concentrations of ROS, they may also have beneficial effects for cellular activity, being involved in several processes such as pathogen elimination, tissue healing, and repair. Furthermore, ROS may also exhibit signaling properties, acting as regulatory factors in many redox-sensitive signaling pathways [5]. While clear beneficial effects of ROS have been shown, a constant balance between ROS production and reduction is imperative. Both enzymatic and non-enzymatic mechanisms are responsible for removing free radicals and inhibiting oxidative processes, by being oxidized themselves [4]. Thus, enzymes such as superoxide dismutases, superoxide reductases, glutathione peroxidase, glutathione reductases, catalase, and heme oxygenase have the useful properties of reducing active ROS to less harmful oxygen species. Furthermore, several endogenous molecules and some vitamins and minerals play important roles, for example, as enzyme cofactors or electron donors [4]. 

A natural balance between ROS generation and degradation is exhibited by most of biological systems involving ROS production and metabolism. The excessive generation of ROS generates an important homeostatic imbalance that eventually leads to many unwanted effects such as oxidative tissue damage and inflammation [4]. While many stressors such as radiation, toxic substance intake, nonsteroidal anti-inflammatory agents, infections, and inflammation may lead to high ROS production, natural antioxidant defenses can limit the harmful effects of them [6]. 

Whereas many of the commonly known exogenous stressors enter the body via the gastrointestinal tract (GIT), pulmonary system, and skin, and only a few studies address other subtypes of stress factor (psychological, congenital, and autoimmune), it is clear that the GIT is a major source of ROS [7]. However, one organ in the human body produces more ROS compared to the GIT: the brain. The body’s first response to high ROS concentrations is a primary inflammatory response, which in fact leads to further ROS production, generating an escalating ROS attack and over-activation of inflammation. In fact, many of the functional GIT pathologies originate from such over responsive oxidative modulation, which is known as oxidative stress [8]. It has been shown that chronic intestinal inflammation is associated with the overproduction of reactive oxygen and nitrogen species (ROS and RNS) [9]. Additionally, some studies have shown that inflammation is generally a key component of the GIT diseases [10,11,12]. While many of the GIT diseases cause or are caused by an exacerbated anti-inflammatory response, it is clear that oxidative stress mechanisms are also impaired. For instance, neutrophil activation requires a reactive potential and therefore an increase in ROS production [13]. In this manner, neutrophil over-activation leads to impaired GIT function [14]. Thus, inflammatory bowel disease and its subtypes, gastrointestinal ulcers and their subtypes, gastroesophageal reflux disease, gastritis, enteritis, colitis, pancreatitis, liver cirrhosis, and cancer in fact originate from oxidative imbalance [15]. 

Withal, it seems that the GIT diseases are the most common systemic impairments occurring in the general population [16]. Their development may involve nutrition, bacterial of viral infections, systemic dysfunction, congenital syndromes, or psychiatric impairment (chronic stress, affective disorders, or neurodegeneration of the intestinal nervous plexus) [17,18]. However, some of the gastrointestinal diseases seem to lack detectable organic causes, being therefore classified as functional disorders [19]. 

## 2. Irritable Bowel Syndrome Pathophysiology and Promoting Factors

Irritable bowel syndrome (IBS), non-erosive reflux disease, and functional dyspepsia are some of the most important functional gastrointestinal syndromes, which can be diagnosed only after excluding all other organic diseases [20]. These syndromes are characterized by gastrointestinal impairments in the absence of a physiological or organic cause followed by inexplicable symptomatology, but with no harmful effects or tissue damage detectable by laboratory testing. In this manner, IBS clearly affects bowel habits, causing pain, constipation or diarrhea, bloating, and gas [21]. Despite the fact that it is now known that there are many factors that make the bowel respond in a such way, IBS treatment is not followed by recovery, but by remission [22].

Although the revised diagnostic criteria guidelines currently include the possible implication of brain gut impairments in IBS symptomatology development [23], the link between oxidative stress and the various types of IBS is not as well documented. The ROME IV criteria classified IBS as diarrhea-predominant IBS (IBS-D), constipation-predominant IBS (IBS-C), mixed stool pattern IBS (IBS-M), and unclassified IBS (IBS-U) [24]. Further classification also includes other criteria such as pain sensation and positive stool culture, generating two new subtypes: pain-predominant IBS and post-infectious IBS (IBS-PI) [25]. Regarding the oxidative stress changes occurring in the different types of IBS, Choghakhori et al. [26] found similar malondyaldehide (MDA) levels, but decreased total antioxidant capacity in IBS-C, IBS-D, and IBS-M. However, the fact that differences occurred in inflammatory markers, such as IL-10, IL-17, and TNFa, in IBS-D but not in other IBS subtypes could lead to the suggestion that the molecular mechanisms underlying the IBS symptomatologies could be rather different. Additionally, their study found mathematical correlations between the immunological, but not biochemical, body of data and pain sensations occurring in IBS. In this context, the flaws in brain-gut signaling could be explained through the serotonin secretion impairments occurring in IBS. In the intestinal tissues, serotonin secretion and release modulates intestinal motility and thus the potential for pain occurrence [27]. Furthermore, Miwa et al. [28] suggested that the impaired release of serotonin is also implicated in IBS-C. However, it is known that diarrhea is a product of exacerbated inflammatory processes, whereas constipation could be the result of slowed motility. In this context, the differences in IBS subtype molecular mechanisms could be due to enterochromaffin cell function being implicated in serotonin modulation, while a more relevant molecular impairment could be altered serotonin receptor activity [27]. Regarding the impairments that occur in the motor function of the GIT, while IBS-D was associated with sympathetic adrenergic dysfunction, IBS-C was described as a parasympathetic dysfunction [29]. In contrast to slow-transit constipation, in which the transit time is prolonged and the high-amplitude propagated contraction frequency is reduced, IBS-typical constipation may not be due to stool transit delay or reduced stool frequency, but due to or accompanied by psychological distress [30]. Thus, this functional constipation usually responds to dietary fiber therapy, not involving other impairments. However, functional constipation may be caused by several neurological disorders and metabolic and endocrine disorders, which may also impair the enteric nervous system (ENS). 

Enteric neuron damage may also be caused by bacterial lipopolysaccharide (found in IBS-PI) and the presence of pro-inflammatory agents, which divert tryptophan (the main serotonin precursor) into the kynurenine pathway, leading to high pro-oxidant agent synthesis [31]. In this manner, with both enteric neuron apoptosis and oxidation-promoted mast cell mutation, a vicious cycle involving an aberrant inflammatory response and gut-brain axis impairment leads to high levels of motilin and serotonin and also extremely high levels of somatostatin [25], which are the main factors regulating bowel motility, therefore resulting in diarrhea. 

The correlation between oxidative status changes in diarrheic syndrome was suggested by Dellan et al. [32], according to which in a lactose-induced diarrhea model, a reduction in vitamin E tissue levels and an increase in the intestinal inflammatory response are often associated with the diarrheic syndrome [32]. Furthermore, both diarrhea and vitamin E deficiency altered lipid peroxidation and primary antioxidant defense systems in various tissues. Similarly, Wang et al. [30] suggested that chronic constipation may lead to oxidative stress and ROS damage due to a significant decrease in vitamin C and vitamin E levels in the plasma, as well as in superoxide dismutase and catalase activity in the same fluid. Also, Li et al. [33] showed that intestinal secretory immunoglobulin A and superoxide dismutase levels decrease, whereas lipid peroxidation increases, in constipated rats. Moreover, Vermorken et al. [34] suggested a correlation between bowel movement frequency, oxidative stress, and several colonic diseases. Thus, the differences between IBS types regarding oxidative stress status could suggest that the molecular mechanisms of IBS symptomatology are rather complex, but modulated by a common pathway.

Consistent evidence suggests that IBS may be associated, in some ways, to depression, anxiety, post-traumatic stress disorder (PTSD), psychological stress, and even dementia [35,36,37,38,39,40]. Furthermore, although IBS coexists with many psychiatric disorders, it was shown that a cause-effect relationship is highly improbable [40] (Table A1). 

Therefore, IBS must be an independent syndrome caused by similar harmful stimuli.

In this context, a correlation between oxidative stress’ effects on the nervous system and IBS would be interesting, given that the nervous system is highly susceptible to oxidative damage due to its particular lipid structures and low antioxidant defenses [41,42,43]. Additionally, several reports show that the implication of oxidative stress in IBS would be probable, but no mechanism is yet clear [44,45,46]. Still, the common ground between some of the mentioned psychiatric illnesses (which are subtypes of affective syndromes) and, according to ROME IV criteria, IBS is the lower or greater extent of stress axis impairment [47]. Previous studies have shown that oxidative stress changes also occurred in stress-based animal models [46,48,49]. This could be a specific piece of evidence that suggests that a central nervous modulation pathway is implicated in IBS pathophysiology, with oxidative stress being a possible signaling pathway but also an unwanted effect. 

Moreover, [50] described a possible pathway of neuroinflammation involved in enteric nerve system impairment that would lead to hyper-excitability followed by impaired intestinal motility. Thus, immune cells such as mast cells or enterochromaffin cells tend to overreact in the colonic mucosa, signaling inflammation to the ENS and further triggering the release of serotonin and several cytokines that mediate local ROS production.

Based on this information, the correlation between IBS and ENS impairment remains to be explained. In spite of the fact that inflammatory bowel disease can be characterized by a clear and positive correlation between oxidative stress, inflammation, and tissue degeneration [51,52], neurosomatic impairment cannot be considered as a plausible cause [50]. By contrast, IBS seems to be less correlated with tissue destruction, oxidative stress, and inflammation and more with ENS impairments [53].

In addition to this aspect, the neuromuscular modulation between the myenteric nerve plexus and the brain to the brainstem is provided by adrenaline and serotonin. Therefore, the communication between CNS and ENS is a two-way road: the enteric system is influenced by the brain, and the brain is influenced through the vagal and sympathetic afferents. Thus, IBS symptomatology may be due to certain dysfunctions in the CNS, gut, or both [39]. 

In addition, several brain imaging studies [54,55] revealed that the visceral stimulation response is actually projected in several brain areas, such as the anterior cingulated cortex, amygdala, insula, and brainstem. In fact, all of these are responsible for emotions, impulse control, fear, pain perception, awareness, and salience [56]. It has been shown that these structures are responsible for producing the actual visceral pain and the corresponding negative emotions common in IBS patients [53]. Therefore, a link between affective or psychiatric disorders and IBS may be explained by these structures’ alteration or a neurotransmitter impairment that could lead both to psychiatric and somatic symptoms. This is the reason why more than 40% of panic disorder patients [57], more than 25% of major depression patients [58], and almost 42% of alcohol abuse or dependence patients [59] also suffer from functional somatic impairments such as intestinal mobility disorders. Less than 20% of schizophrenia patients also meet the criteria for IBS [60].

Thus, IBS seems to be determined by imperfect CNS gastrointestinal nervous system modulation. Whereas in IBS, no changes in intestinal cell consistency have been reported [61], neurovisceral disorders impairing the brain-gut axis may partially explain the neurosomatic features of IBS. Thus, in the absence of nutritional or inflammatory stimuli, inflammation and oxidative stress do not significantly damage gut tissues; they may be also considered as collateral effects of impaired neurostimulation [50].

Similarly, several studies have suggested sex and gender-related differences in IBS symptomatology development. Thus, several studies point to a gender difference in IBS. In industrialized countries, it has been observed that it is predominately women that seek health care services during acute or chronic symptomatology, as compared with men [62]. Drossman et al. [63] reported a 2:1 ratio (men/women) in IBS frequency. However, some studies [64,65] suggest that only regarding IBS-C is responsible for the gender differences, whereas [66] report that women predominantly exhibit IBS-C symptoms and most men have the IBS-D subtype. Since it has been suggested that the causes leading to IBS are more likely to be heterogeneous, most of the patients find that diet and stress aggravated IBS, and menstrual cycle fluctuations are frequently reported as a related symptom [63]. Therefore, female gender may influence the symptomatology of IBS since it seems that a higher frequency of female patients has been recorded [67].

Supporting this hypothesis, [68] showed that besides the clear implication of nutritional habits, IBS may be also triggered by hormonal disturbances in women. It has been shown that local estrogen administration in the rat hippocampus inhibits serotonin re-uptake [69] and increases serotonin assimilation in tandem with lower cerebrospinal fluid (CSF) serotonin levels [70]. Therefore, high estrogen levels may influence the functionality of the serotonergic intestinal plexus synapses and enterochromatoffin cell serotonin release, causing altered intestinal motility. Additionally, some studies clearly showed that intestinal infections may lead to a seven-fold increase in IBS occurrence [18,27,71]. Ji et al. [71] and Mearin et al. [72] showed that IBS is more likely to occur in bacterial gastroenteritis patients (*Shigella* sp., and, respectively, *Salmonella* sp.) than in healthy subjects. Moreover, Wang et al. [18] and Okhuysen et al. [72] refer to bacillary dysentery and bacterial diarrhea as some of the most important factors that can lead to IBS. Moreover, it seems that several changes produced by bacterial infections could persist after healing. Therefore, while bacterial infections lead to strong inflammatory responses accompanied by high ROS production, a correlation between the remaining inflammatory changes and IBS context fulfilment could be proposed [73].

In this context, aside from the role of pathogenic bacteria, the role of the microbiota was studied in the context of IBS. Considering the active implication of the gut microbiota for body homeostasis and human behavior [74,75], the “second brain” from the gut was found to be the missing link in the communication between the brain and the gut. Balmus et al. [76], Mari et al. [77] and Actis et al. [78] offered a thorough description of the implication of the gut microbiota in IBS and in bowel inflammatory disorders. 

## 3. Neuroendocrine Alterations in Irritable Bowel Syndrome 

As previously described, the brain-gut axis seems to exhibit several changes in IBS patients. Recently, Wouters et al. [79] described bowel motor impairment, visceral hypersensitivity, or the abnormal processing of sensations in IBS patients. Considering these, a new pathological approach was possible by concluding that IBS may be, in fact, a brain-gut axis disorder, as newly described by the ROME IV diagnosis criteria for functional gastrointestinal disorders. Furthermore, Lembo and Bollom [80] characterized a new IBS type, psychiatric impairment-associated IBS, revealing that IBS may also exhibit typical psychosomatic symptomatology (leading to the emergence of a new IBS type based on the impairment in pain sensation, pain predominant IBS). 

Interestingly enough, some reports also showed that central nervous system excitants such as coffee and chocolate may also trigger IBS development [81,82,83,84]. Due to the fact that caffeine is a strong adenosine antagonist possessing the capacity to block all of the adenosine receptors [85], it could modulate intestinal inflammation through parasympathetic signaling. In this manner, it seems that caffeine, and also theophylline, antagonism promotes neurotransmitter release, such as the release of acetylcholine, which endows the stimulant effects [86]. Furthermore, over-stimulation can sometimes produce inflammatory responses, leading to the assumption that CNS excitants may in fact, act peripherally as inflammatory modulators.

Moreover, oxidative stress may be implicated in motor nerve function due to a ROS-mediated decrease in cholesterol synthesis (vital to myelin formation). Therefore, the enteric nerve plexus should not present any exception. While changes generated by oxidative stress to the peripheral nervous system are clearly possible—and have been demonstrated to be the cause of peripheral nerve degeneration [87]—and IBS clearly exhibits a neurological component represented by the gut-brain axis [88], it is possible that the effects of oxidative stress could be correlated to several IBS symptoms.

Furthermore, Kennedy et al. [89] showed that IBS may be correlated with brain impairments affecting cognitive performance measured by visuospatial memory accuracy. In this manner, it seems that the well-known theory which links IBS with hypothalamic–pituitary–adrenal (HPA) axis dysfunction is now further demonstrated by the correlation between low cortisol levels and hippocampus-mediated memory performance. Moreover, Kennedy et al. also suggested that both amygdala-mediated emotional cognitive alterations and non-emotional hippocampus-mediated visuospatial episodic memory alterations may occur in IBS. Additionally, the unique IBS pain perception relies on altered visceral pain perception since the anterior cingulate cortex is not affected at the cognitive level. In 2014, the same group [90] also suggeste bidirectional brain-gut impairment in IBS. Based on the microbiome activity and function in neuroendocrine regulation, they show that microbiome imbalance, which is thought to lead to PI-IBS, may actually create an imbalance in the HPA-axis through an elevated immune response. Taken together with the fact that many mood disorder patients also present IBS-like GIT impairments [91], it could be hypothesized that IBS may actually be a two-way brain-gut axis impairment. Moreover, a large academic study [92] clearly points to visible brain changes in IBS patients. 

Also, the correlation between brain and gut neurological communication can be demonstrated by various neurological disorders that clearly exhibit functional GIT effects similarly to IBS. For instance, Chapman et al. [93] points to a direct correlation between comorbid psychiatric disorders and the functional GIT symptomatology found in mitochondrial disease. Furthermore, Perkin and Murray-Lyon [94] extensively discussed the implications of neurological diseases in GIT impairments. In some cases, IBS-like symptomatology has been reported in fibromyalgia and chronic pain disorders [95,96]. Similar abdominal pain episodes have been reported, as described in pain predominant-IBS. Additionally, several mood disorders commonly exhibit IBS-like intestinal symptomatology due to brain-gut-brain impairment [39,97]. It has been shown that depression, anxiety, ataxia, and attention deficit hyperactivity disorder may exhibit strong GIT manifestations [97]. Moreover, the symptomatology of neuropsychiatric disorders such as schizophrenia, autism, and peripheral neuropathy may include IBS-like features [98]. Also, Catassi [98] described gluten sensitivity as a both intestinal and neurological impairment. One atypical example of a GIT symptom of a neurological disorder is the abdominal chronic pain observed in syringomyelia [99], which is due to cerebrospinal fluid overflow in the abdominal cavity. In addition, it seems that other chronic pain conditions are accompanied by IBS, predominantly in female patients [39]. 

On the other hand, it seems that some GIT disorders closely resemble IBS symptomatology. A few research groups have also suggested that IBS and inflammatory bowel disease (IBD) are, in fact, interrelated [100], since the remission of IBD is almost always an IBS-like experience [101]. Thus, IBS brain stimulation clearly focuses on pain perception and emotional responses to pain given by the activation of the limbic system. By contrast, IBD might not necessarily cause an afferent impulse. Therefore, it seems that a correlation between IBD and the brain-gut axis may be less significant, due to the fact that IBD is an organic disease. Moreover, the extreme inflammatory response to intestinal damage and ROS accumulation may also lead to enteric nervous system impairment alongside intestinal cells’ mass apoptosis [51].

## 4. Irritable Bowel Syndrome, Oxidative Stress, and Inflammation

Although it is theoretically stated that no immunological and histological similarities between IBD and IBS can be highlighted, Barbara et al. [102] clearly showed in 2002 that low grade intestinal inflammation could occur in IBS. Therefore, the aforementioned group reported that some patients with IBS exhibit an increase in inflammatory colonic and ileal mucosa cells that may be due to infectious enteritis, undiagnosed food allergies, genetic factors, and changes in the bacterial microflora. They also stated that minimal grade inflammation may perturb gastrointestinal reflexes and activate the visceral sensory system leading to abnormal neuro-immune interactions and the altered gastrointestinal physiology and hypersensitivity underlying IBS. Based on intestinal inflammation animal models, it has been shown that IBS-PI could be caused by intestinal muscle dysfunction and muscularis externa inflammation [103,104,105].

Moreover, the presence of active immune cells in the intestinal mucosa of the ileum and colon in the IBS patients was previously documented [106,107,108]. Although Ahn et al. [109] showed important changes in cellular inflammatory markers (mast cells increased by 97.6%, intra-epithelial lymphocytes increased by 92.8%, and lamina propria lymphocytes increased by 81.9%), Theoharides [110] points to the imminent observation that despite the number of inflammatory cells possibly being increased, it is their activation that should lead to conclusions. However, it was highlighted that the stress induced by increased mast cells led to intestinal permeabilization that could not necessarily be correlated to IBS occurrence [110].

Additionally, it has been shown that more than one fourth of infectious enteritis patients develop IBS-PI. The fact that more than 70% of acute enteritis patients do not further develop IBS [111] reveals that IBS may be, in fact, a multi-factorial syndrome that requires certain risk factors.

It seems that the key role in the inflammatory response is played by the mast cells, which also are located close to enteric nerves modulating gut-nervous system communication, by releasing many inflammatory mediators capable of affecting enteric nerve function and muscle contractility [112]. Furthermore, while nitric oxide is a key modulator of mast cell activation and also a signaling molecule in the nervous system, it seems that mast cell-synthesized nitric oxide may also be involved in IBS. Thus, O’Sullivan et al. [113] found increased an expression of inducible nitric oxide synthase and high levels of nitric oxide in IBS patients’ colons.

In addition, an interesting genetic study revealed that an IBS genetic predisposition regarding the gut inflammatory status is possible. In order to compare IBD and IBS inflammatory potential, Chan et al. [114] tested the expression of two major anti-inflammatory agents, IL-10 and TGF-beta. It was shown that both anti-inflammatory agents’ expression was reduced in IBS patients, leading to the idea that anti-inflammatory cytokine production may protect against IBS. Thereby, it seems that a genetic predisposition to low anti-inflammatory agent production [114] may be one of the ways through which inflammation occurs in IBS. Furthermore, a correlation between stress and colonic inflammation was described due to the activation of mast cells [115] and even the reactivation of previous inflammation [116] after exposure to high stress conditions.

Since several GIT disorders involving altered inflammatory responses that cause oxidative attacks on intestinal cellular layers may also exhibit some IBS-like symptoms, a connection between IBS, inflammation, and oxidative stress may be explained through the possible predisposition of IBS patients to IBD [117]. Additionally, a correlation between the gut-brain axis and IBD was observed [118].

However, Bernstein et al. [119] described several afferent signaling modulation systems in IBD patients, as compared to in IBS patients. Therefore, it has been shown that sensory processing is impaired in both IBD and IBS. The difference could rely on pain perception, for example. It seems that brain stimulation caused by pain is differently localized in IBS and IBD patients’ brains. Furthermore, it was demonstrated that chronic colonic inflammation may not be correlated to afferent signaling during rectal distension [20] and that limbic systems are obviously activated in IBS, but inactive in IBD [120].

On the one hand, IBD is also characterized by increased oxidative stress followed by intestinal cells layer damage via free radical-dependent apoptosis [43]. Previously, diet and stress were thought to be decisive factors in IBD occurrence, but it is now clear that IBD is more likely to be linked to heredity, immune system malfunction, or aberrant responses to intestinal bacterial or viral infections [121,122].

In fact, our group also showed that active IBD patients exhibit an increased activity of superoxide dismutase and glutathione peroxidase, as well as significant MDA synthesis versus controls [52]. Contrarily, remission patients had significantly lower SOD and GPx activities and also increased lipid peroxidation. Due to high oxidative stress, IBD may be characterized by consistent intestinal cellular layer damage and immune response malfunction. Moreover, control patients did not exhibit any oxidative stress marker changes. Therefore, it is possible that stress or diet would not influence the mechanisms by which IBD is triggered [52].

Furthermore, oxidative stress may be an important IBS pathological factor, since tissue damage due to oxidative attack is currently associated with inflammation [52]. Several studies associate mild inflammation with IBS [102,103,104,105,106,107]. Barbara et al. [102] suggests that low grade inflammation plays a role in IBS sensorimotor dysfunction. Thus, the increased inflammatory cell distribution may help explain the regional differences in colonic motor dysfunction or visceral hypersensitivity. Eriksson et al. [123] showed some minor differences in C-peptide, triglyceride, prolactine, and cortisol levels by comparing IBS-C, IBS-D, and IBS-M, but with no statistical significance. While mast cell and macrophage activation and pro-inflammatory agent release involve oxidative status changes, low levels of oxidative stress should also occur. Thus, the presence and activation of infiltrated mast cells would imply oxidative stress occurrence, which would be detectable [124].

The observation that some of patients with acute gastroenteritis developed IBS seems also to be a matter of inflammation [125]. Moreover, several human studies revealed that myeloperoxidase activity increases, alongside the expression of tumor necrosis factor alpha and production of interleukin 1b and interleukin 6 [125,126]. Similarly, previous studies showed that inflammation may also occur as a result of hormonal stress modulation. Dinan et al. [127] showed that plasma levels of IL-6 are increased in IBS patients but TNF-α levels were normal, meaning that common organic inflammation would not be a characteristic of IBS. Interestingly, they also showed that IBS inflammation is positively correlated with adrenocorticotropic hormone levels, suggesting that the correlation between stress hormones and inflammation could be causative or determinant. In other words, the inflammation occurring in IBS could be modulated by adenocorticotropic hormone, or vice versa.

Based on these observations, Hong-Yan Qin et al. [128] suggested that psychological stress, alongside the manifestation of anxiety (independently or as a result of stress exposure), could lead to important changes in the inflammatory profiles of IBS patients. At the same time, they also observed that one of these processes could cause dysbiosis. Furthermore, Mozaffari et al. [46] theorized that an increased inflammatory response may imply high oxidative status in correlation with neuropsychiatric implications, by observing behavior and oxidative and inflammatory profiles in a wrap-restraint rat model. The fact that these results were obtained in the absence of an organic cause may point to stronger implications of the hypothalamic-pituitary-adrenal axis. However, it is not clear which of the two association partners (neuropsychiatric or gastroenterological symptoms) is the cause. Therefore, it would be possible that IBS could also be correlated with neuropsychiatric impairments, even if not categorized as a neuropsychiatric impairment with clinical gastrointestinal symptomatology.

Besides the obvious inflammatory response stimulation as discussed above, stress has been shown to increase intestinal membrane permeability, allowing inflammatory signals to pass through the membrane and lead to inflammatory cell accumulation in the muscularis and lamina propria. Furthermore, some authors [46,52,129] correlated inflammatory cell accumulation with increased oxidative stress. 

In order to further explore this potential IBS characteristic, Mete et al. [50] evaluated the oxidative status in 36 IBS patients. Malondyaldehide (MDA), oxidant and antioxidant enzymes, and nitric oxide concentrations were assessed in the blood serum. In this manner, plasma xanthine oxidase activity was found to be significantly increased in IBS patients, as compared to in controls. Since xanthine oxidase plays an important role in inflammation through its capacity to produce reactive oxygen species (hydroxyl radical), IBS inflammation may be positively correlated with xanthine oxidase pro-oxidant activity. 

However, regarding the implication of oxidative stress in IBS as a permanent component alongside inflammation, there are still unsolved issues. On the one hand, Mozaffari et al. [46] showed that decreased antioxidant capacity occurs during IBS development. On the other hand, Covarrubias et al. [130] showed an increase in antioxidant system expression as a result of positive feedback to high ROS production. Therefore, it is clearly possible that a certain mechanism is producing high levels of ROS that, even with an over-expression of the antioxidant system, could not be counteracted. Thus, the existence of a ROS-producing mechanism that is more powerful than the antioxidant system in an over-expression state could be possible.

The connections between oxidative stress and IBS were also confirmed by Oran et al. [131], who evaluated paraoxanase and arylesterase activities in IBS patients. It seems that these two enzymes work together in forming the antioxidant enzymatic complex needed in anti-inflammatory response modulation. Still, based on the gender differences previously described in the current literature, Oran et al. reported no significant difference in terms of age and gender between the groups. In fact, neither Mete et al. [50] nor, more recently, Karakas et al. [132] considered separate groups based on gender when assessing oxidative stress effects. 

Furthermore, oxidative stress and inflammation occurrence in IBS could be explained through tryptophan metabolism impairments [45]. This mechanism would be relevant in the sense that indolamine deoxygenase is known to catalyze the oxidation of tryptophan to kynurenine by producing high concentrations of hydrogen peroxide.

**Figure 1:**
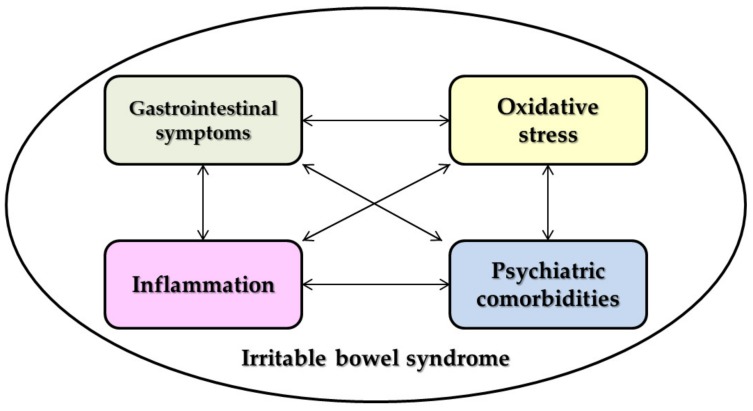
The interconnection between the irritable bowel syndrome (IBS) pathophysiological components.

Thus, the complex connections between IBS and oxidative stress, most of the neuropsychiatric disorders, inflammation and the gastrointestinal symptoms are summarized in Figure 1.

## 5. Conclusions

In accordance with the current knowledge on IBS pathophysiology and mechanism of occurrence, the molecular mechanisms, such as inflammation and oxidative stress, as well as stress modulation pathways, could be closely related to the development of the gastrointestinal symptoms of IBS. In this context, the involvement of the neurological structures in the absence of any tissue-based damage could lead to the assumption that the mechanics of IBS include a strong neuropsychiatric component. Additionally, although mild inflammation and therefore mild oxidative stress may occur in IBS, the main impairment that decisively contributes to IBS pathology may be gut-brain axis bidirectional communication errors. Therefore, it seems likely that there is a possible correlation between neurological impairment, emotional over-responsiveness, mild inflammatory patterns, and oxidative stress, which are not followed by tissue destruction in IBS patients. 

## Appendix A

**Table A1:** Psychiatric comorbidities reported in IBS patients (diarrhea-predominant IBS (IBS-D), constipation-predominant IBS (IBS-C), mixed stool pattern IBS (IBS-M), unclassified IBS (IBS-U), post-infectious IBS (IBS-PI)).

**IBS-C**	Generalized anxiety [133]	Depressive episodes [133]	Sleep impairments [134]	Somatisation [133]	Alcohol abuse [135]	Migraine [134]	Anger [136]	Psychosis [137]	Paranoid ideation [137]	Phobic anxiety [137]	
**IBS-D**	Generalized anxiety [133]	Depressive episodes [133]	Migraine [138]	Somatisation [133]	Sleep impairments [134]						
**IBS-M**	Generalized anxiety [133]	Depressive episodes [133]	Mixed anxiety/depressive episodes [133]	Sleep impairments [134]	Somatisation [133]	OCD [133]	PTSD [133]	Migraine [134]	Panic disorder [133]	Adjustment disorder [133]	Dysthymia [133]
**IBS-PI**	Anxiety [139]	Depression [139]	Neuroticism [139]	Somatisation [139]	Distress [139]	Negative perception of illness [138]	Hypochondriasis [140]	Sleep disorders [141]			
**IBS-U**	Anxiety [142]	Depression [142]	Distress [142]	Sleep impairments [134]	Migraine [134]						
